# Supplementary material for: An evolutionarily young defense metabolite influences the root growth of plants via the ancient TOR signaling pathway
Source: eLife. 2017 Dec 12;6:e29353. doi: 10.7554/eLife.29353 (PMC5730369; doi:10.7554/eLife.29353)
Supplement: Supplementary file 1. — The source, common name, order, family, genus, species, and subspecies seeds are listed for the used plant species. As well as the plate size (cm × cm), and plating distance used for the individual response assays. [file elife-29353-supp1.docx]

| **Common name** | **Order** | **Family** | **Genus** | **Species** | **Subspecies** | **Supplier/Donor** | **Plate size (cm×cm)** | **Distance to top (cm)** | **Distance between seeds (cm)** |
| --- | --- | --- | --- | --- | --- | --- | --- | --- | --- |
| Dill | Apiales | Apicaceae | Anethum | Graveolens | Mammut | Albertines A/S (Føtex) | 12×12 | 6 | 2 |
| Garden cress | Brassicales | Brassicaceae | Lepedium | Sativum | - | Albertines A/S (Føtex) | 24×24 | 7 | 3 |
| Rape seed | Brassicales | Brassicaceae | Brassica | Napus | - | a gift (see acknowledgements) | 24×24 | 5 | 3 |
| Rucola | Brassicales | Brassicaceae | Eruca | Sativa | Rouqette | Albertines A/S (Føtex) | 12×12 | 3 | 2 |
| Broccoli | Brassicales | Brassicaceae | Brassica | Oleraceae | Italica | Albertines A/S (Føtex) | 24×24 | 7 | 4 |
| Camelina | Brassicales | Brassicaceae | Camelina | Sativa | - | was a gift (see acknowledgements) | 24×24 | 5 | 3 |
| Lotus | Fabales | Fabaceae | Lotus | Japonicus | MG20 | a gift (see acknowledgements) | 12×12 | 6 | 2 |
| Oregano | Lamiales | Lamiaceae | Origanum | vulgare | - | Albertines A/S (Føtex) | 12×12 | 3 | 1 |
| Tomato | Solanales | Solanaceae | Solanum | Lycopersicum | San marzano | Niels Møller Rasmussens Gartneri I/S  (Kvickly) | 24×24 | 7 | 2 |
| Flax | Malpighiales | Linaceae | Linum | usitatissimum | - | Urtekram ® International A/S, Midsona AB (Føtex) | 24×24 | 7 | 3 |

**Sup. File 1. Plant seeds used for *in vitro* root growth assays for the various plant species**

The, source, common name, order, family, genus, species, and subspecies seeds are listed for the used plant species. As well as the plate size (cm×cm), and plating distance used for the individual response assays.
