# Supplementary material for: An evolutionarily young defense metabolite influences the root growth of plants via the ancient TOR signaling pathway
Source: eLife. 2017 Dec 12;6:e29353. doi: 10.7554/eLife.29353 (PMC5730369; doi:10.7554/eLife.29353)
Supplement: Transparent reporting form [file elife-29353-transrepform.docx]

***eLife’s* transparent reporting form**

We encourage authors to provide detailed information *within their submission* to facilitate the interpretation and replication of experiments. If you have any questions, please contact us: [editorial@elifesciences.org](mailto:editorial@elifesciences.org).

**Sample-size estimation**

- You should state whether an appropriate sample size was computed when the study was being designed
- You should state the statistical method of sample size computation and any required assumptions
- If no explicit power analysis was used, you should describe how you decided what sample (replicate) size (number) to use

Please outline where this information can be found within the submission (e.g., page numbers or figure legends), or explain why this information doesn’t apply to your submission:

The information regarding sample sizes, number of replications and the applied statistical methods is indicated in the individual figure legends. This averaged 10 or more independent biological replicates in 3 or more experiments for a standard of 30 independent replicates for each genotype x treatment. This is typically sufficient to provide significant statistical power for even 5% effect sizes in our conditions and techniques.

Tor further boost the power, all the data and replicates for each analysis was combined in single linear models that incorporate experimental effects to better contrast the biological factors.

**Replicates**

- You should report how often each experiment was performed
- You should include a definition of biological versus technical replication
- The data obtained should be provided and sufficient information should be provided to indicate the number of independent biological and/or technical replicates
- If you encountered any outliers, you should describe how these were handled
- Criteria for exclusion/inclusion of data should be clearly stated
- High-throughput sequence data should be uploaded before submission, with a private link for reviewers provided (these are available from both GEO and ArrayExpress)

Please outline where this information can be found within the submission (e.g., page numbers or figure legends), or explain why this information doesn’t apply to your submission:

Throughout the manuscript, the number of replications within each experiment (n) and experimental replicates are indicated in the individual figure legends. All data has been included into the analysis by combining the experiments in ANOVAs.

**Statistical reporting**

- Statistical analysis methods should be described and justified
- Raw data should be presented in figures whenever informative to do so (typically when N per group is less than 10)
- For each experiment, you should identify the statistical tests used, exact values of N, definitions of center, methods of multiple test correction, and dispersion and precision measures (e.g., mean, median, SD, SEM, confidence intervals; and, for the major substantive results, a measure of effect size (e.g., Pearson's r, Cohen's d)
- Report exact p-values wherever possible alongside the summary statistics and 95% confidence intervals. These should be reported for all key questions and not only when the p-value is less than 0.05.

The number of replications within each experiment (n) and the statistical tests utilized are indicated in the individual figure legends. The p values are shown in the figures.

Raw data is not presented in the figures as dots because N is almost always greater than 10 and the data is always from 3 or more independent experiments that are not properly shown as distributions of raw data.

Please outline where this information can be found within the submission (e.g., page numbers or figure legends), or explain why this information doesn’t apply to your submission:

(For large datasets, or papers with a very large number of statistical tests, you may upload a single table file with tests, Ns, etc., with reference to page numbers in the manuscript.)

**Additional data files (“source data”)**

- We encourage you to upload relevant additional data files, such as numerical data that are represented as a graph in a figure, or as a summary table
- Where provided, these should be in the most useful format, and they can be uploaded as “Source data” files linked to a main figure or table
- Include model definition files including the full list of parameters used
- Include code used for data analysis (e.g., R, MatLab)
- Avoid stating that data files are “available upon request”

Please indicate the figures or tables for which source data files have been provided:
